# Assessing health risks of complementary alternative medicines in cancer patients

**DOI:** 10.1038/sj.bjc.6602044

**Published:** 2004-07-13

**Authors:** G Shia, D Shaw, P I Dargan

**Affiliations:** 1 Chinese Medicine Advisory Service, NHS Trust Medical Toxicology Unit, Guy's and St Thomas' Hospital, Avonley Road, London SW14 5ER, UK

**Sir,**

Werneke *et al* (2004) highlighted several important issues in their article. They confirmed the findings of other authors (Ernst and Cassileth, 1998; Newell and Sanson-Fisher, 2000) that herbal supplements are commonly used among cancer patients, but often unsupervised. Health care professionals have expressed concerns about their safety and possible interaction with pharmaceutical drugs. The authors suggested a joint medicines information and toxicology service to address such concerns. Such a service already exists.

The Medical Toxicology Unit, Guy's & St Thomas' Hospital Trust, has been providing information on herbal medicines to health care professionals for over 10 years. As well as providing safety information, we also assist in reviewing the suspected adverse health effects of these medicines. This work led to the development of the Chinese Medicine Advisory Service in 2001, which specialises in providing information to health care professionals on issues surrounding the use of Chinese herbal medicines by their patients. We would like to add some of our findings and experience to those published in the article.

A herb is deemed potentially toxic either because it contains pharmacologically active constituents, or if the plant extract has been shown to be toxic in animal studies or if there have been previous case reports of toxicity associated with its use. Among these three sources we feel that, generally, high-quality clinical case reports are the most informative. We scrutinise each report to establish its causal relationship and the circumstances of poisoning, considering factors such as whether the toxicity was dose related or due to inappropriate use. From our work, we have found that most adverse effects arise from human errors such as overdosage or poor quality control.

For any enquiry, we try to take into account the potential benefits of the ingredients. We investigate such claims with bibliographical evidence. Practising herbalists also inform us of their empirical experience. We arrive at a pragmatic decision and our advice is based on the balance between risks and benefits of the ingredients.

Potential herb–drug interactions is an area where it can be more difficult to give balanced answers at present. This is because most of the suspicions are extrapolated from what is known about the pharmacology of the herb or its constituents, and not from actual clinical events. This is also a major problem with drug–drug interactions, as was clearly expressed by Stockley (2002), ‘The data on interactions are of widely varying quality and reliability. Sometimes they are no more than speculative and theoretical scaremongering guesswork, hallowed by repeated quotation until they become virtually set in stone’. This is not to suggest that potential herb–drug interactions should be ignored, rather it illustrates the need for further investigations to be able to identify combinations in which interactions are likely to result in significant adverse effects. The significance of drug–herb interaction in cancer treatment can only be properly evaluated by a prospective study of patients taking herbal medicine in addition to conventional therapy and evaluating the benefits and adverse effects due to their interaction. Having carried out this initial survey of patients, Dr Werneke and the team are well placed to be able to carry out such a prospective study of their patients, and show the clinical relevance of the suspected interactions.

Until further data are available, our current practice in making recommendation with regard to possible herb–drug interactions is to suggest closer monitoring of the patient, and we advise the physician on the area that needs to be focused. For instance, in a patient who takes warfarin, we would normally recommend more frequent measurement of the INR for a period of time after the introduction of a new herbal agent.

There is some evidence suggesting that the supplementary use of herbal medicines can have additional beneficial effects. An *in vitro* study has shown that gamolenic acid (oil from the seeds of evening primrose and borage) potentiates the cytotoxicity of paclitaxel and vinorelbime in human breast cancer cell lines (Menendez *et al*, 2002). There are a number of clinical trials from China that suggested that Chinese herbal medicines used together with conventional therapies improve the mortality and morbidity of cancer patients (Liu *et al*, 2001; Liang *et al*, 2003). These findings were often substantiated by experimental studies (Liu *et al*, 2002; Qu *et al*, 2003). These trials will need to be confirmed independently, but nevertheless suggest interesting synergism.

Currently, the amount of evidence based on local data is limited. Between 1999 and 2002, the CSM/MHRA received 345 herbal adverse reaction reports; of these 40% related to St John's Wort (Barnes, 2003). Improved awareness of and reporting suspected herbal adverse effects and interactions are clearly required. Doctors should take a full drug history from their patients, including traditional medicines and herbal products. The World Health Organisation has emphasised the importance of exchanging knowledge through an international network to overcome shortage of information from own region (WHO Traditional Medicine Strategy, 2002–2005, 2002). We believe that multiple reference sources, especially those published from countries where herbal medicine is more commonly used, should be used in order to provide an evidence-based advice.
